# Accuracy of Pulse Wave Velocity Predicting Cardiovascular and All-Cause Mortality. A Systematic Review and Meta-Analysis

**DOI:** 10.3390/jcm9072080

**Published:** 2020-07-02

**Authors:** Irene Sequí-Domínguez, Iván Cavero-Redondo, Celia Álvarez-Bueno, Diana P Pozuelo-Carrascosa, Sergio Nuñez de Arenas-Arroyo, Vicente Martínez-Vizcaíno

**Affiliations:** 1 Universidad de Castilla-La Mancha, Health and Social Research Center, 16071 Cuenca, Spain; Irene.Sequi@alu.uclm.es (I.S.-D.); Celia.AlvarezBueno@uclm.es (C.Á.-B.); DianaP.Pozuelo@uclm.es (D.P.P.-C.); Sergio.NunezdeArenas@uclm.es (S.N.d.A.-A.); Vicente.Martinez@uclm.es (V.M.-V.); 2 Universidad Politécnica y Artística del Paraguay, Asunción 001518, Paraguay; 3 Faculty of Physiotherapy and Nursing of Toledo, University of Castilla-La Mancha, 45005 Toledo, Spain; 4 Multidisciplinary Research Group in Care (IMCU), University of Castilla-La Mancha, 45005 Toledo, Spain; 5 Facultad de Ciencias de la Salud, Universidad Autónoma de Chile, Talca 3467987, Chile

**Keywords:** arterial stiffness, pulse wave velocity, predictive performance, cardiovascular risk, cardiovascular mortality, all-cause mortality

## Abstract

Increased arterial stiffness has been associated with an increased risk of developing cardiovascular diseases and all-cause mortality. Pulse wave velocity (PWV) is an innovative and affordable measurement of arterial stiffness which may be an accessible tool to estimate mortality risk; however, no meta-analysis has estimated its predictive performance for cardiovascular and all-cause mortality. Moreover, reference values for PWV have only been established by consensus for healthy populations. The aim of this review was to estimate PWV and especially carotid femoral PWV performance predicting cardiovascular and all-cause mortality as well as comparing the resulting cfPWV thresholds with already established values in order to increase its validity. Original studies measuring PWV thresholds and its association with cardiovascular and all-cause mortality were systematically searched. The DerSimonian and Laird method was used to compute pooled estimates of diagnostic odds ratio (dOR), and overall test performances were summarized in hierarchical summary receiver operating characteristic curves (HSROC). Six studies were included in the meta-analysis. The pooled dOR values for the predictive performance of cfPWV were 11.23 (95 % CI, 7.29–1.29) for cardiovascular mortality and 6.52 (95% CI, 4.03–10.55) for all-cause mortality. The area under the HSROC curve for cfPWV was 0.75 (95% CI, 0.69–0.81) for cardiovascular mortality and 0.78 (95% CI, 0.74–0.83) for all-cause mortality, where the closest cut-off point to the summary point was 10.7 and 11.5, respectively. This systematic review and meta-analysis demonstrates that cfPWV is a useful and accurate cardiovascular mortality predictor and that its previously estimated reference values for estimating risk may be used in high-risk populations.

## 1. Introduction

Vascular ageing measurements are presumed to be a useful tool to estimate cardiovascular risk. Increased arterial stiffness, defined as the reduced ability of an artery to expand and contract in response to pressure changes [1], has been associated with the development of cardiovascular disease [2,3]. Arterial stiffening induces an early return of arterial wave reflection consequently increasing systolic blood pressure (SBP), while reducing diastolic blood pressure (DBP). This causes an increased left ventricular afterload and altered coronary perfusion [4]. Moreover, high blood pressure induces vascular ageing by causing chronic arterial inflammation and diffuses intima-media thickening, and as such, changes in blood pressure can be considered both a cause and a consequence of arterial stiffness [5]. Nevertheless, arterial stiffening is not only an atherosclerosis-related outcome [6], but also a consequence of the exposure to many contributing factors, such as age, hypertension or diabetes [7].

Arterial stiffness can be measured through non-invasive, reproducible, and relatively inexpensive techniques, such as the measurement of pulse wave velocity (PWV) [8]. PWV is considered the gold standard method for assessing aortic stiffness [9,10]. Arterial stiffness measures, and carotid femoral PWV (cfPWV) in particular, are being included in the routine clinical assessment of patients and within the framework of large-scale clinical studies [9] as new instrumental solutions that allow the PWV assessment, such as photoplethysmography or magnetic resonance emerge [11] (Table 1). Nevertheless, an introduction into clinical practice has not been implemented further due to the fact that there is a lack of established reference values based on a large population and due to the absence of a standardized methodology for PWV assessment [12].

Previous meta-analyses have attempted to calculate quantitative estimates of the predictive value of PWV for different outcomes. However, to the best of our knowledge, no previous meta-analysis has estimated the predictive performance (diagnostic odds ratio (dOR), sensitivity, specificity, positive likelihood ratio (PLR), and negative likelihood ratio (NLR)) of PWV considering the thresholds for a higher risk of cardiovascular or all-cause mortality estimated using hierarchical summary receiver operating characteristic (HSROC) models. Moreover, reference values for PWV have been established through cross-sectional studies [12] or expert consensus [13], in which subjects by age and blood pressure categories with no additional identified cardiovascular risk factors were considered. However, as these reference values are not yet fully incorporated into clinical practice, there is uncertainty regarding whether such values are applicable to high-risk subjects.

Therefore, the aim of this systematic review and meta-analysis was to estimate the predictive performance of PWV for cardiovascular and all-cause mortality using an HSROC analysis as well as comparing the resulting PWV thresholds with those established in order to improve its validity.

## 2. Methods

This systematic review and meta-analysis was reported following the Preferred Reporting Items for Systematic Reviews of Interventions (PRISMA) statement [14] and the recommendations of the Cochrane Handbook for Systematic Reviews of Diagnostic Test Accuracy [15]. This study was registered in the PROSPERO International Prospective Register of Systematic Reviews (registration number: CRD42018080949).

### 2.1. Literature Search

PubMed (via MEDLINE), EMBASE (via Scopus) and Web of Science databases were searched systematically from inception to June 2020. The following free-terms were included in the search strategy combined with Boolean operators following the PICO strategy: “arterial stiffness”, stiffness, “pulse wave velocity”, PWV, “aortic pulse wave velocity”, “carotid-femoral pulse wave velocity”, “brachial-ankle pulse wave velocity”, cfPWV, baPWV, predict*, marker, “cut-off”, prognostic, “cut-point”, sensitivity, specificity, threshold, mortality, death, “cardiovascular mortality”, “cardiovascular risk”, “all-cause mortality”, “cardiac death” or survival (Figure S1).

### 2.2. Selection Criteria

Eligible articles were original studies measuring PWV thresholds and their association with cardiovascular and all-cause mortality, that is, PWV predictive performance. Thus, inclusion criteria were as follows: (i) study participants aged ≥18 years; (ii) measured PWV (brachial-ankle PWV (baPWV) or carotid-femoral PWV (cfPWV)); (iii) study design: longitudinal studies with prospective or retrospective data collection; and (iv) reported sensitivity, specificity and 2 × 2 table. Studies were excluded if they: (i) were not written in English or Spanish; and ii) did not report cardiovascular or all-cause mortality as an outcome.

### 2.3. Data Extraction and Quality Assessment

The following data was extracted from each included study: (i) author identification and year of publication; (ii) country of study; (iii) characteristics of the population; (iv) age of the participants; (v) number of participants; (vi) number of deaths (cardiovascular and/or all-cause); (vii) PWV test used; and (viii) parameters summarizing the accuracy of the test (cut-off point, sensitivity, specificity, area under curve (AUC) and diagnostic odds ratio (dOR)).

The Quality in Prognosis Studies (QUIPS) tool for studies of prognostic factors [16] was used to assess the risk of bias of each included study. This tool evaluates six bias domains: study participation, study attrition, prognostic factor measurement, outcome measurement, study confounding, statistical analysis, and reporting. Each may be rated as having high, moderate or low risk of bias.

The literature search, data extraction, and quality assessment were performed by two independent researchers (IS-D and IC-R). Inconsistencies were solved by consensus.

### 2.4. Statistical Analysis and Data Synthesis

The sensitivity, specificity, PLR, NLR, AUC, and dOR along with their corresponding 95% confidence intervals (CIs) were calculated for the PWV test used in each included study.

HSROC curves were used to summarize overall test performance as multivariate methods that jointly analyze sensitivity and specificity. These curves have been proposed to estimate the diagnostic performance of tests in meta-analyses, where the prediction region is useful for estimating the magnitude of heterogeneity in such a way that wider prediction regions suggest greater heterogeneity [17]. An AUC closer to one indicates a more accurate test, that is the probability of a randomly selected pair of a true-positive and a true-negative being ranked as such by the diagnostic test, an AUC value of less than 0.75 may be reasonable, though it indicates weaknesses in the test accuracy [18].

The dOR is a measure of the accuracy of a diagnostic test that combines sensitivity and specificity into a single value. The value ranges from zero to infinity, with a value of one corresponding to a null diagnostic ability and a higher value to a better discriminatory test performance. The dOR was computed using Moses’ constant of a linear model. This approach relies on the linear regression of the logarithm of the dOR of a study as a dependent variable and on an expression of the positivity threshold of a study as an independent variable [19].

The DerSimonian and Laird method [20] was used to compute pooled estimates of dOR for each included study. The heterogeneity of results across studies was assessed using the statistical parameter I2 and the corresponding p values. I2 values were considered as follows: might not be important (0–30%); may represent moderate heterogeneity (30–50%); substantial heterogeneity (50–75%); and considerable heterogeneity (75–100%) [21].

Sensitivity analyses were performed to estimate the individual influence of each particular study in the pooled dOR by removing studies one by one. Subgroup analyses were performed for cardiovascular mortality and all-cause mortality. Random-effects meta-regression models were used to evaluate if the cut-off points for PWV values and the mean age and percentage of women of participants influenced dOR values. Finally, publication bias was evaluated by visually examining funnel plots and through Deeks’ method [22].

All statistical analyses were performed using STATA SE software, version 15 (StataCorp, College Station, TX, USA).

## 3. Results

### 3.1. Baseline Characteristics

**Figure 1:**
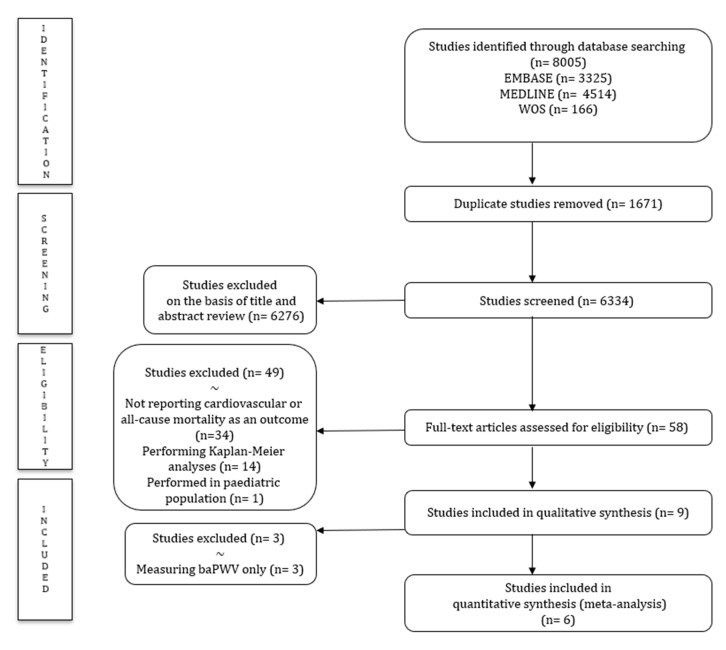
Preferred Reporting Items for Systematic Reviews and Meta-Analyses (PRISMA) diagram of the systematic literature search strategy.

After removing duplicates, the titles and abstracts of 58 articles were screened. Following the full-text review, nine studies were included in this systematic review, and due to the scarcity of studies measuring baPWV, only studies measuring cfPWV [6,7,23,24,25,26] were included in the main quantitative data synthesis (Figure 1).

All included studies were of longitudinal nature [6,24,25,26,27,28,29], two of them were cross-sectional analyses from longitudinal studies [7,23]. Studies were published between 1999 and 2014 and were performed in six different countries: France [23,24,25], Japan [27,28], Korea [29], Macedonia [6], Portugal [7], and the United States [26].

A total of 3170 participants were included with mean ages ranging from 53.1 to 76.4 years. Studies were carried out in specific populations, such as patients undergoing dialysis [6,7], hypertensive patients [23,27], end-stage renal failure patients [24,25], elderly population [28], patients who had a percutaneous coronary intervention [29], who can all be considered high-risk populations, and the general population [26] (Table 2).

### 3.2. Risk of Bias

As evaluated with the QUIPS tool, all studies provided information regarding the six quality domains. Most studies had shortcomings in the study participation and study confounding domains (55.5% and 77.8% of studies scored as moderate risk of bias, respectively). An overall moderate bias risk was obtained since the weakest quality study only had three domains with a moderate score (Table S1).

### 3.3. Meta-Analysis

**Figure 2:**
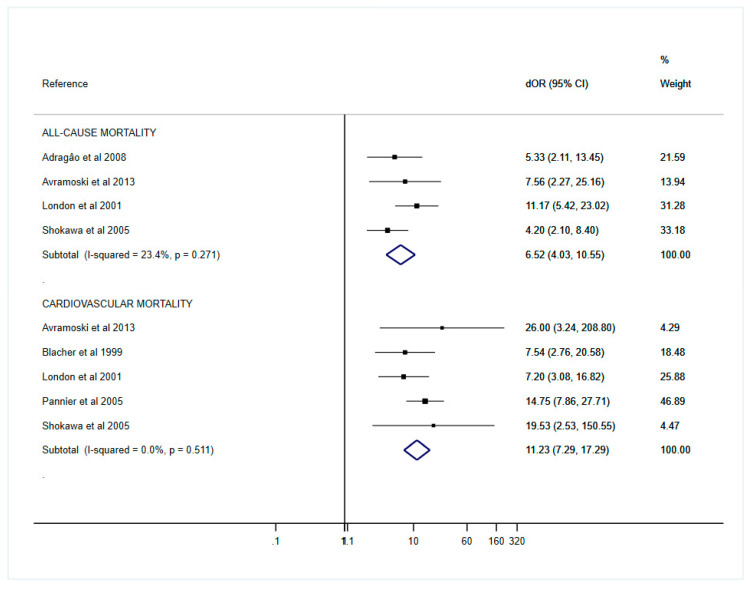
Forest plot of pooled dOR estimates for all-cause and cardiovascular mortality.

A meta-analysis was only performed for cfPWV, as the main analysis, due to the small number of included studies using baPWV (three studies). Although both are measures of arterial stiffness, baPWV measures the stiffness of peripheral arteries, while cfPWV is an indicator of stiffness of central elastic arteries, and therefore, their thresholds differ markedly because of their different measurement points; however, a meta-analysis was performed for baPWV despite the scarcity of studies (Figure S2). The pooled dORs for cardiovascular mortality were 11.23 (95% CI, 7.29–17.29) and 6.52 (95% CI, 4.03–10.55) for all-cause mortality. No important heterogeneity across studies was found in the cfPWV dOR for cardiovascular mortality (I^2^ = 0.0%, *p* = 0.511) or all-cause mortality (I^2^ = 23.4%, *p* = 0.271), as can be observed in the forest plots (Figure 2). The pooled sensitivity, specificity, PLR, NLR, dOR and AUC are presented in Table 3.

In order to avoid potential bias, we performed a meta-analysis that excluded the only study performed in the general population with slightly smaller pooled dORs for cardiovascular mortality 9.08 (95% CI, 4.31–13.84) and slightly higher dORs for all-cause mortality 7.12 (95% CI, 2.72–11.52), with no important heterogeneity across studies found for cardiovascular mortality (I^2^ = 0.0%, *p* = 0.62) or all-cause mortality (I^2^ = 0.0%, *p* = 0.55).

**Figure 3:**
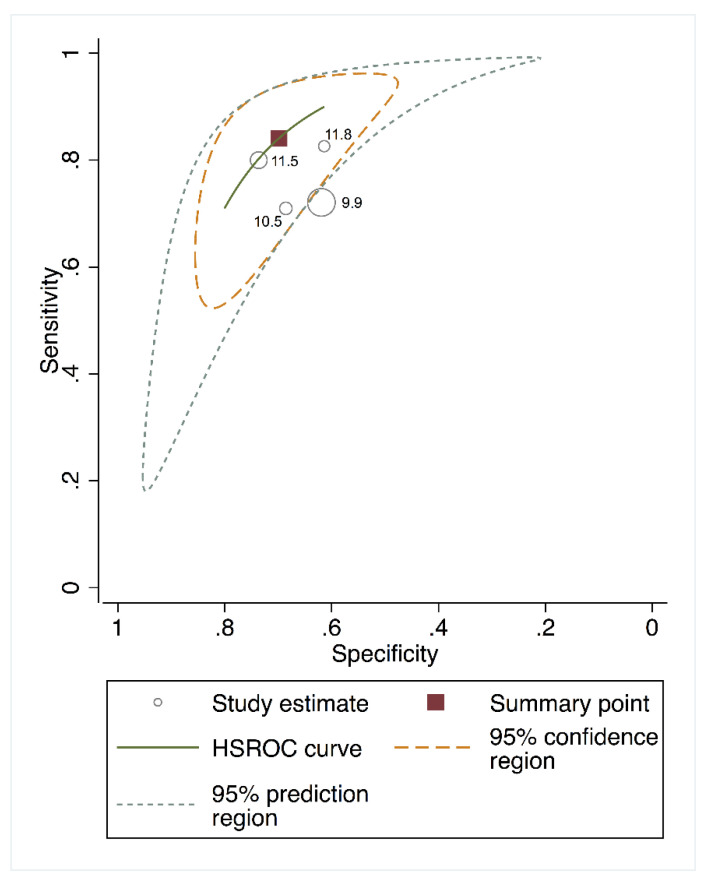
Hierarchical summary receiver operating characteristic curves (HSROC) curve for cfPWV predicting all-cause mortality.

**Figure 4:**
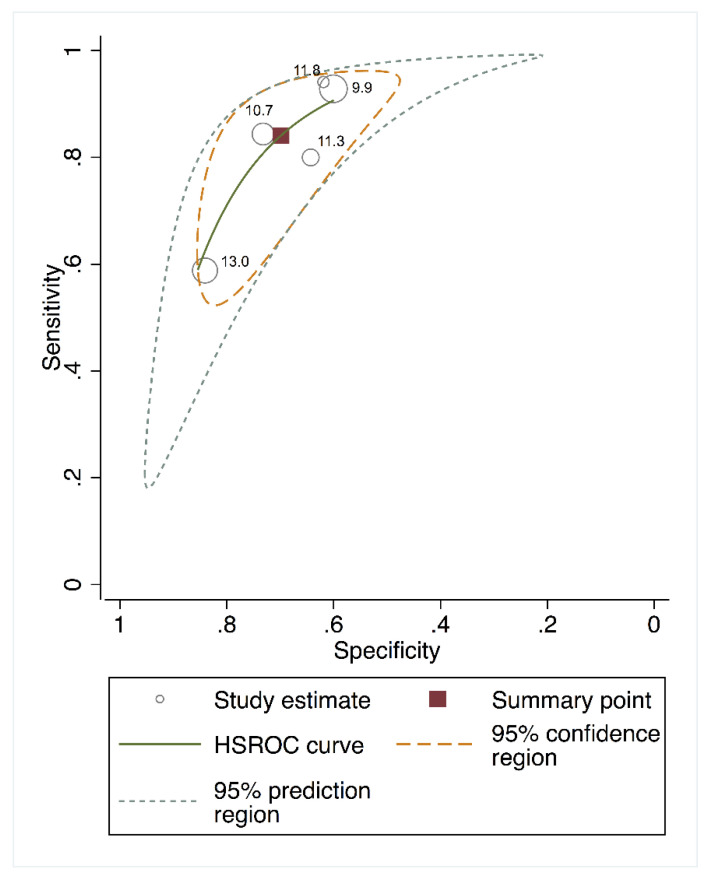
HSROC curve for cfPWV predicting cardiovascular mortality.

The area under the HSROC curve for estimating the predictive performance of cfPWV was 0.78 (95% CI, 0.740–0.830) for cardiovascular mortality and 0.75 (95% CI, 0.690–0.810) for all-cause mortality. The 95% confidence region for the point estimate that summarized the overall test performance in the area under the HSROC curve (Figure 3 and Figure 4) included studies in which the test cut-offs ranged from 9.9 to 13.0 m/s for cardiovascular mortality and from 9.9 to 11.8 m/s for all-cause mortality. The closest cut-off point to the summary point was 10.7 for cardiovascular mortality and 11.5 for all-cause mortality, as shown in Figure 3 and Figure 4, respectively.

### 3.4. Sensitivity Analyses for the Effect of Individual Studies

The pooled dOR estimates for cfPWV were not affected after studies were removed one at a time from the analyses to evaluate their individual impact on cardiovascular and all-cause mortality.

### 3.5. Random Effects Meta-Regression Model

Although random effects meta-regression models are only recommended for meta-analysis including ten or more studies [30], they were used to determine whether the cfPWV cut-off points were related to the dORs for cardiovascular (*p* = 0.354) and all-cause mortality (*p* = 0.210), concluding that there was no statistically significant effect (Figures S5 and S6). Moreover, there was no statistically significant results between age and dOR estimates for neither cardiovascular (*p* = 0.995) nor all-cause mortality (*p* = 0.208) (Figures S7 and S8) or between the percentage of women and dOR estimates for cardiovascular (*p* = 0.85) or all-cause mortality (*p* = 0.59) (Figures S9 and S10).

### 3.6. Publication Bias

Deeks’ funnel plot for asymmetry suggested the absence of publication bias for cardiovascular and all-cause mortality (*p* = 0.890 and *p* = 0.850, respectively) (Figures S11 and S12).

## 4. Discussion

Arterial stiffness measurements are becoming a field of interest as previous evidence has showed their role as an independent risk predictor for cardiovascular disease [31]. However, to the best of our knowledge, no previous study has estimated the predictive performance of PWV for cardiovascular and all-cause mortality. Our results indicate a good accuracy of cfPWV for cardiovascular mortality (dOR: 11.23 (95% CI, 7.29–17.29); sensitivity: 83% (95% CI, 71.00–79.00); specificity: 71% (95% CI, 66.00–75.00)), and slightly lower accuracy values for all-cause mortality. Furthermore, data regarding the cfPWV AUC for cardiovascular mortality showed good accuracy levels.

Previous meta-analyses [8,32] have provided evidence on the predictive value of PWV for cardiovascular events and all-cause mortality, thereby demonstrating the importance of arterial stiffness as an indicator of cardiovascular risk. Our results do not only support previous findings, but also estimate the threshold that represents the range of increased risk.

cfPWV reference values have been previously defined as 10 m/s through consensus [13] or based on large cross-sectional studies [12] focused mainly on healthy populations. Our results support such recommendations even for high-risk populations, as the cut-off points in the current study ranged between 9.9 and 13 for cardiovascular mortality, and from 9.9 to 11.8 for all-cause mortality. The smallest cut-off point was obtained from the only study performed in the general population [26] and is probably due to the narrower threshold of all-cause mortality than the higher risk population samples. However, it should be noted that the measurement of PWV is of particular clinical interest in improving the predictive ability of cardiovascular risk in intermediate risk patients [33]; thus, in order to provide additional evidence supporting the inclusion on this new biomarker to improve the risk stratification and, consequently, tailoring more precisely the treatment of patients, more randomized clinical trials are needed.

cfPWV is the recommended arterial stiffness measurement method according to the American Heart Association (AHA) scientific statement [10], the European expert consensus document [34] and European Society of Cardiology (ESC) and the European Society of Hypertension (ESH) Guidelines for the management of arterial hypertension [35] due to the large preponderance of longitudinal data from cohort studies. However, as is common with rapidly developing technologies, the standardization of techniques is required [10]. Six of the studies included in this review measured cfPWV, [6,7,23,24,25,26] and three measured baPWV [27,28,29], in fact, the latter is not as established as cfPWV. In our systematic review and meta-analysis, predictive performance measures remarkably decreased when cfPWV was analyzed in combination with baPWV (Figures S3 and S4 and Table S2). This may be attributable to the differences between function and structure of central elastic arteries (cfPWV), whose main function is to maintain a relatively constant pressure gradient despite the constant pumping action of the heart, and stiffer peripheral muscular arteries with predominantly conduit function (baPWV) [36]. Therefore, there is a dilemma between the higher accuracy of cfPWV and the measuring easiness of baPWV since the latter only requires the wrapping of blood pressure cuffs on the four limbs [37], nevertheless such dilemmas may be dissipated as new PWV measuring methods are developed, such as oscillometric methods or photoplethysmography, with additional functionality and greater ease-of-use, presenting this methods as a more applicable tool for PWV assessment in daily clinical practice [11,38].

Vascular biomarkers, such as ankle-brachial index, arterial stiffness, endothelial function, and circulating biomarkers related to vascular wall structure have been suggested for risk assessment in prevention strategies of cardiovascular events [39]. PWV, as a measure of arterial stiffness, has been proven to be effective in predicting the risk of cardiovascular events and mortality as well as all-cause mortality. However, the relevance of PWV to traditional risk scores has not been clearly examined. Several studies, such as Rhee et al. for Framingham risk score [40] or Pereira et al. for HeartSCORE [41], have evaluated the benefits of integrating PWV in cardiovascular risk assessment strategies to improve their discriminative capacity. Therefore, a new scenario has emerged which, despite requiring further research, may lead to the inclusion of vascular health measurements, such as PWV, in cardiovascular risk scores.

This systematic review and meta-analysis has some potential limitations to be acknowledged: (i) despite the lack of clear evidence of publication bias, studies with a poor test performance may be less or more likely to be published; (ii) some studies could not have been included because they were published in languages other than English or Spanish, or were grey literature (PhD dissertations, institutional reports, etc.); (iii) from 8005 studies retrieved by the search strategy, only nine referred to studies examining or reporting harder endpoints (cardiovascular and all-cause mortality), the scarcity of studies including predictive performance measurements and cardiovascular events as an outcome, means that further research is needed; (iv) included studies were performed in specific populations, such as elderly people, patients undergoing dialysis or hypertensive patients, making it difficult to infer our results to the general population; (v) not all of the included studies performed cfPWV measurements using the same technique or the same device, which may bias the estimates obtained, also taking into account the high intra-individual variation of this measurement (which could be due to biological variability/measurement error). However, despite the previously mentioned limitations, our results may lead to further research, which may establish cfPWV as an accurate risk predictor of cardiovascular and all-cause mortality, and consequently justifying future research and its inclusion in daily clinical practice, which, as long as it requires a great deal of effort from clinicians and health systems to implement it, strong evidence is needed to endorse such change.

## 5. Conclusions

cfPWV constitutes a good cardiovascular and all-cause mortality predictor, since it has been shown to have good accuracy in estimating cardiovascular and all-cause mortality risk, although its accuracy was observed to be much higher for cardiovascular mortality. Consequently, cfPWV constitutes a feasible, non-invasive and replicable method for estimating risk, and enabling its use in high-risk populations. Moreover, our data confirm that the cfPWV cut-off values previously established by scientific societies are applicable to high-risk populations.

Nevertheless, further research is necessary to decrease the impact of the limitations of the current review and extend the results obtained by studying PWV’s predictive ability in the general population and if it may extend beyond cardiovascular events.

## Figures and Tables

**Table 1:** Methods used to determine PWV. aPWV: aortic pulse wave velocity; baPWV: brachial-ankle pulse wave velocity; cfPWV: carotid-femoral pulse wave velocity; DVP: digital volume pulse; ECG: electrocardiogram; PWV: pulse wave velocity.

	Method	Description	Measure
Non-invasive methods	Applanation tonometry	Apply a pressure sensor through the skin and applanate a superficial artery by applying a downward pressure sufficient to flatten the artery.	baPWV, cfPWV
	Computerized oscillometry	Simultaneous acquisition and analysis of the pulsation of the artery, which is caused by the heart, as the pressure oscillation in the cuff.	Heart-brachial PWV, heart-ankle PWV, brachial-ankle PWV, cfPWV
	Mechanotransducer	Two dedicated piezoelectric pressure mechanotransducers directly applied to the skin in a simultaneous measurement of pressure pulses	carotid–femoral, carotid–brachial or femoral–dorsalis pedis PWV
	Ultrasound	Doppler pulses are recorded sequentially in 2 different arterial sites and compared using the R-wave of the ECG	baPWV, cfPWV
	Photoplethysmography	DVP measured by the photoplethysmography transducer	DVP associated with aPWV
	Magnetic Resonance Imaging	Assessment of the blood flow velocity with an enough temporal and spatial resolution to study the propagation of the aortic systolic flow wave	Local PWV
Invasive methods	Aortic angiography	Intra-aortic catheter measurements	Local PWV

**Table 2:** Characteristics of studies included in the meta-analysis.

Author	Country	Population	Age	n (% Female)	n Mortality	Index Test (Device)	Cut-Off Point	Sens (%)	Spec (%)	AUC	dOR
Adragão et al. 2008	Portugal	Dialysis patients	58.9	101 (29.7)	All-cause: 31	cfPWV (Complior)	10.5	71.0	69.0	0.738	5.33
Avramoski et al. 2013	Macedonia	Dialysis patients	61.3	80 (33.75)	All-cause: 23	cfPWV (pulsed-Doppler ultrasound synchronized with ECG)	11.8	82.6	61.4	0.722	7.56
					CV: 17		11.8	94.1	61.4	0.820	26.00
Blacher et al. 1999	France	Hypertensive patients	62.0	710 (41.8)	CV: NA	cfPWV (Complior)	13	60.0	84.0	0.780	7.54
Kawai et al. 2012	Japan	Hypertensive patients	61.0	400 (45.5)	All-cause: 17	baPWV (FCP-4731)	18	71.0	71.0	0.719	5.88
London et al. 2001	France	End-stage renal failure patients	54.0	180 (40)	All-cause: 70	cfPWV (SPT-301)	11.5	80.0	74.0	0.820	11.17
					CV: 40		11.3	79.0	64.0	0.760	7.20
Miyano et al. 2010	Japan	Elderly population	76.4	530 (31)	All-cause: 30	baPWV (BP-203I)	19.6	73.0	63.0	0.673	4.68
					CV: 11		19.6	91.0	62.0	0.795	16.34
Pannier et al. 2005	France	End-stage renal failure patients	53.1	305 (38)	CV: 96	cfPWV (SEGA M842 8MHz Doppler unit and Gould 8188 recorder)	10.7	84.0	73.0	0.834	14.75
Seo et al. 2014	Korea	Post-percutaneous coronary intervention patients	65.2	372 (36.8)	CV: 21	baPWV (BP-203RPE II)	16.7	85.7	60.1	0.778	9.04
Shokawa et al. 2005	USA/Japan	General population	64.5	492 (55.3)	All-cause: 43	cfPWV (MCG400)	9.9	72.0	62.0	0.690	4.20
					CV: 14		9.9	93.0	60.0	0.770	19.53

Sens: sensitivity; Spec: specificity; AUC: area under curve; dOR: diagnostic odds ratio; CV: cardiovascular; ECG: electrocardiogram; NA: not available.

**Table 3:** Pooled accuracy parameters in the prediction of mortality (cfPWV).

	Sensitivity (%)	Specificity (%)	PLR	NLR	dOR	AUC
**All-cause mortality**	77.00 (65.00–91.00)	65.00 (59.00–71.00)	2.33 (0.66–8.19)	0.34 (0.09–1.25)	6.50 (4.30–9.83)	0.750 (0.690–0.810)
**CV mortality**	83.00 (71.00–97.00)	71.00 (66.00–75.00)	2.68 (0.90–8.00)	0.21 (0.07–0.65)	11.23 (7.29–17.29)	0.780 (0.740–0.830)

Values in parentheses are 95% confidence intervals. CV: cardiovascular; PLR: positive likelihood ratio; NLR: negative likelihood ratio; dOR: diagnostic odds ratio; AUC: area under curve.

## Supplementary Materials

The following are available online at https://www.mdpi.com/2077-0383/9/7/2080/s1. Figure S1. PICO search strategy, Figure S2. Forest plot of pooled dOR estimates for baPWV for all-cause and cardiovascular mortality, Figure S3. HSROC curve for PWV (combining cfPWV and baPWV values) predicting cardiovascular mortality, Figure S4. HSROC curve for PWV (combining cfPWV and baPWV values) predicting all-cause mortality, Figure S5. Meta-regression of cfPWV cut-off points for cardiovascular mortality, Figure S6. Meta-regression of cfPWV cut-off points for all-cause mortality, Figure S7. Meta-regression of age for cardiovascular mortality, Figure S8. Meta-regression of age for all-cause mortality, Figure S9. Meta-regression of % female for cardiovascular mortality, Figure S10. Meta-regression of % female for all-cause mortality, Figure S11. Deeks’ funnel plot graph of cfPWV and cardiovascular mortality, Figure S12. Deeks’ funnel plot graph of cfPWV and all-cause mortality, Supplementary Table S1, and Supplementary Table S2.
