# Supplementary material for: Accuracy of Pulse Wave Velocity Predicting Cardiovascular and All-Cause Mortality. A Systematic Review and Meta-Analysis
Source: J Clin Med. 2020 Jul 2;9(7):2080. doi: 10.3390/jcm9072080 (PMC7408852; doi:10.3390/jcm9072080)
Supplement: Supplementary file 1 [file jcm-09-02080-s001.zip › Supplementary Table S1.pdf]

Table S1. Quality of studies assessed by the QUIPS tool.

	RISK OF BIAS					
	**Study Participation**	**Study Attrition**	**Prognostic Factor Measurement**	**Outcome Measurement**	**Study Confounding**	**Statistical Analysis and Reporting**
**ADRAGÃO ET AL 2008**	Moderate	Low	Low	Low	Moderate	Low
**AVRAMOSKI ET AL 2013**	Low	Low	Low	Low	Moderate	Low
**BLACHER ET AL 1999**	Low	Low	Low	Low	Moderate	Low
**KAWAI ET AL 2012**	Moderate	Moderate	Low	Low	Moderate	Low
**LONDON ET AL 2001**	Moderate	Low	Low	Low	Moderate	Low
**MIYANO ET AL 2010**	Moderate	Low	Low	Low	Low	Low
**PANNIER ET AL 2005**	Moderate	Low	Low	Low	Moderate	Low
**SEO ET AL 2014**	Low	Moderate	Low	Low	Moderate	Low
**SHOKAWA ET AL 2005**	Low	Low	Low	Low	Low	Low
