# Supplementary material for: Accuracy of Pulse Wave Velocity Predicting Cardiovascular and All-Cause Mortality. A Systematic Review and Meta-Analysis
Source: J Clin Med. 2020 Jul 2;9(7):2080. doi: 10.3390/jcm9072080 (PMC7408852; doi:10.3390/jcm9072080)
Supplement: Supplementary file 1 [file jcm-09-02080-s001.zip › Supplementary Table S2.pdf]

Table S2. Pooled accuracy parameters in the prediction of mortality (PWV, combining baPWV and cfPWV.

.	Sensitivity (%)	Specificity (%)	PLR	NLR	dOR	AUC
**All-cause mortality**	75.80 (65.00–88.40)	65.80 (61.90–69.90)	2.29 (0.81–6.43)	0.37 (0.13–1.05)	6.07 (4.28 – 8.60)	0.740 (0.710 -0.780
**CV mortality**	83.50 (72.20–96.70)	66.90 (63.60–70.50)	2.55 (1.02–6.42)	0.20 (0.08–0.53)	11.14 (7.47 – 16.61)	0.780 (0.750 -0.820

Values in parentheses are 95%confidence intervals. CV: cardiovascular, PLR: positive likelihood ratio, NLR: negative likelihood ratio, dOR: diagnostic odds ratio, AUC: Area under curve.
