# Supplementary figures and images for: Accuracy of Pulse Wave Velocity Predicting Cardiovascular and All-Cause Mortality. A Systematic Review and Meta-Analysis
Source: J Clin Med. 2020 Jul 2;9(7):2080. doi: 10.3390/jcm9072080 (PMC7408852; doi:10.3390/jcm9072080)

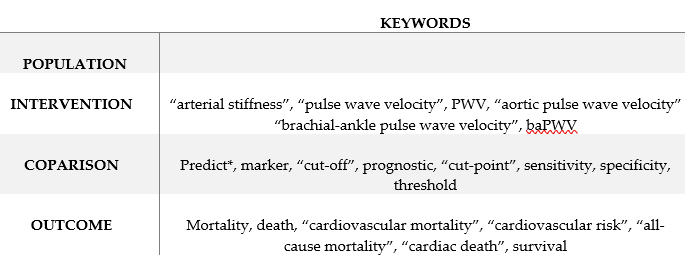

Supplement: Supplementary file 1 [file jcm-09-02080-s001.zip › Figure S1. PICO search strategy..png]

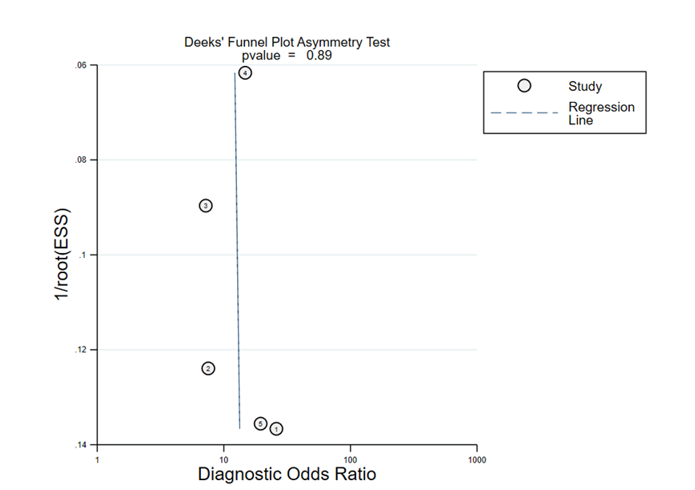

Supplement: Supplementary file 1 [file jcm-09-02080-s001.zip › Figure S10. Deeks Funnel Plot graph of cfPWV and cardiovascular mortality..png]

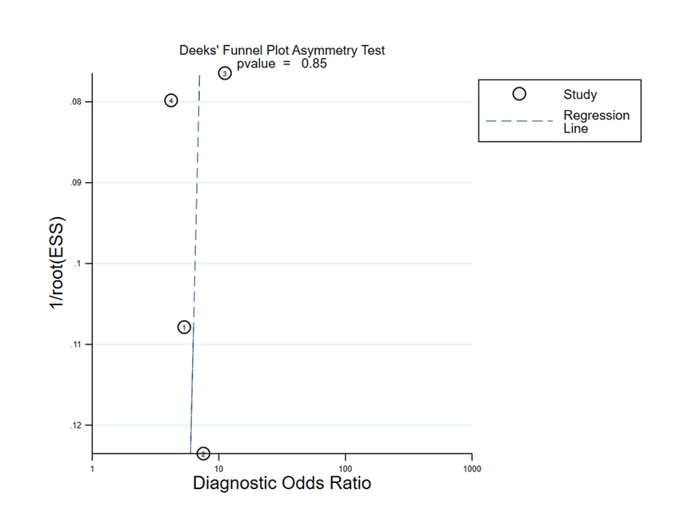

Supplement: Supplementary file 1 [file jcm-09-02080-s001.zip › Figure S11. Deeks Funnel Plot graph of cfPWV and all-cause mortality..png]

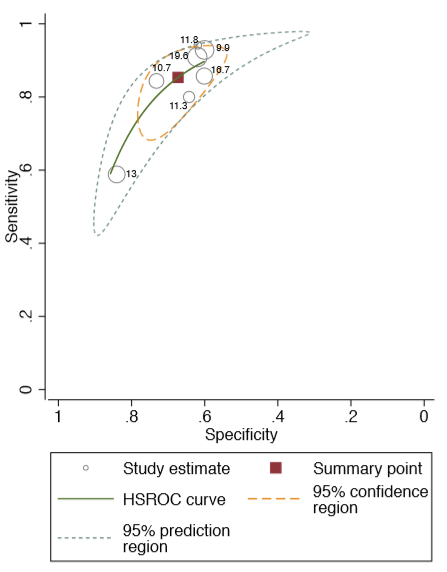

Supplement: Supplementary file 1 [file jcm-09-02080-s001.zip › Figure S2. HSROC curve for PWV (combining cfPWV and baPWV values) predicting cardiovascular mortality.png]

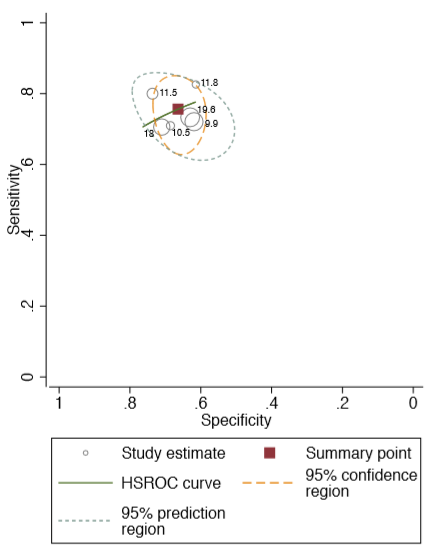

Supplement: Supplementary file 1 [file jcm-09-02080-s001.zip › Figure S3. HSROC curve for PWV (combining cfPWV and baPWV values) predicting all-cause mortality..png]

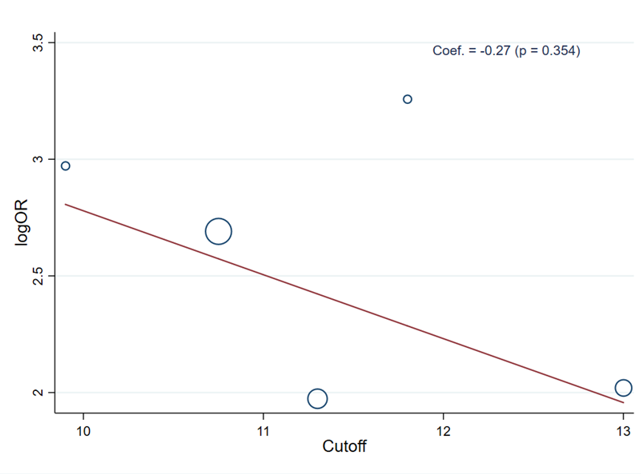

Supplement: Supplementary file 1 [file jcm-09-02080-s001.zip › Figure S4. Meta regression of cfPWV cut-off points for cardiovascular mortality..png]

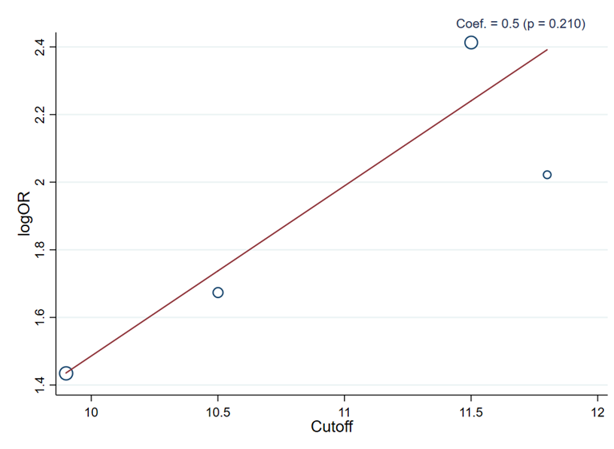

Supplement: Supplementary file 1 [file jcm-09-02080-s001.zip › Figure S5. Meta regression of cfPWV cut-off points for all-cause mortality..png]

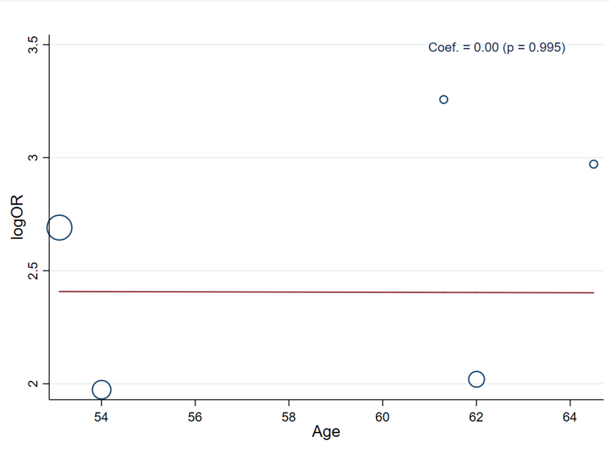

Supplement: Supplementary file 1 [file jcm-09-02080-s001.zip › Figure S6. Meta-regression of age for cardiovascular mortality..png]

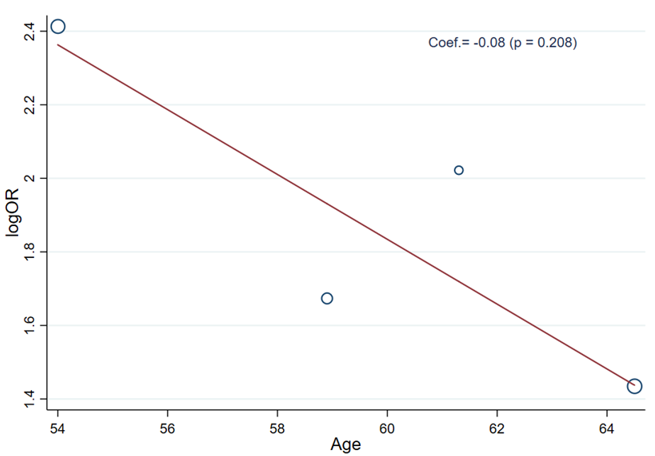

Supplement: Supplementary file 1 [file jcm-09-02080-s001.zip › Figure S7. Meta-regression of age for all-cause mortality..png]

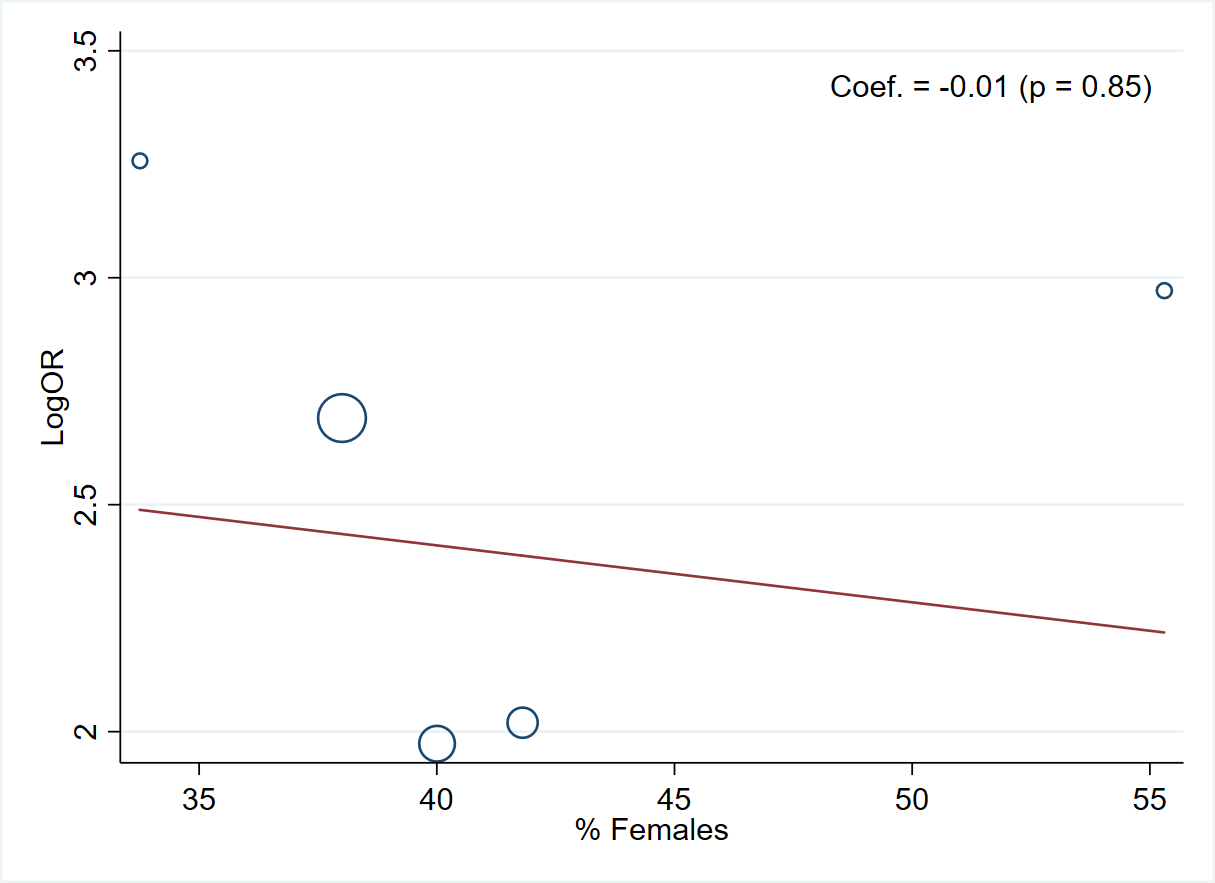

Supplement: Supplementary file 1 [file jcm-09-02080-s001.zip › Figure S8. Meta-regression of % female for cardiovascular mortality..tif]

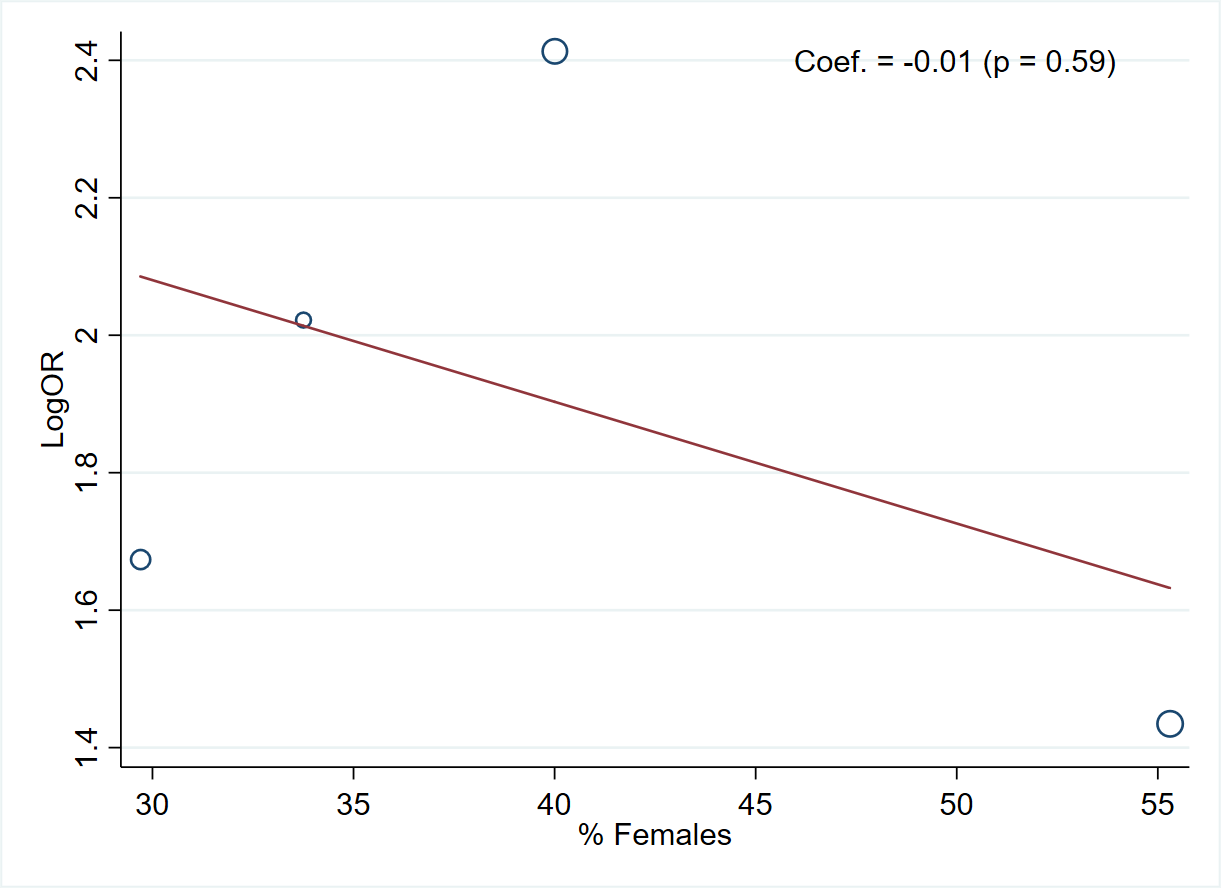

Supplement: Supplementary file 1 [file jcm-09-02080-s001.zip › Figure S9. Meta-regression of % female for all-cause mortality..tif]
